# Electroacupuncture improves cognitive impairment in diabetic cognitive dysfunction rats by regulating the mitochondrial autophagy pathway

**DOI:** 10.1186/s12576-022-00854-0

**Published:** 2022-11-22

**Authors:** Xia Ge, Ling Wang, Qianqian Cui, Hongli Yan, Zhongbao Wang, Shandong Ye, Qingping Zhang, Aihua Fei

**Affiliations:** 1 Department of Endocrinology, The Second Affiliated Hospital of Anhui University of Chinese Medicine, Hefei, 230001 China; 2 College of Second Clinical Medical, Anhui University of Chinese Medicine, Hefei, 230012 China; 3 Department of Emergency, The Second Affiliated Hospital of Anhui University of Chinese Medicine, Hefei, 230001 China; 4 Department of Acupuncture-Moxibustion and Rehabilitation, Mingguang Hospital of Traditional Chinese Medicine, Chuzhou, 239499 China; 5 College of Third Clinical Medical, Anhui University of Chinese Medicine, Hefei, 230012 China; 6 Department of Endocrinology, The First Affiliated Hospital of University of Science and Technology of China, Hefei, 230002 China; 7 College of Acupuncture-Moxibustion and Tuina, Anhui University of Chinese Medicine, Hefei, 230012 China

**Keywords:** Diabetes-associated cognitive impairment, Electroacupuncture, Mitophagy, DISC1

## Abstract

**Background:**

Diabetes-associated cognitive dysfunction has become a major public health concern. However, the mechanisms driving this disease are elusive. Herein, we explored how electroacupuncture improves learning and memory function in diabetic rats.

**Methods:**

The diabetic model was established by intraperitoneal injection of streptozotocin (STZ) in adult Sprague–Dawley rats. Rats were fed on high-fat and high-sugar diets. Learning and memory functions were assessed using behavioral tests. The hematoxylin and eosin (H&E) staining, Western blotting, real-time PCR, ELISA, immunohistochemistry, and transmission electronic microscopy (TEM) was performed to test related indicators.

**Results:**

High-fat and high-sugar diets impaired learning and memory function in rats, while electroacupuncture treatment reversed these changes. The model group presented highly prolonged escape latency compared to the control group, indicating impaired learning and memory functions. The TEM examination showed that electroacupuncture enhanced Aβ clearance and mitochondrial autophagy in hippocampal neuronal cells by increasing DISC1 expression.

**Conclusions:**

Electroacupuncture improves learning and memory function in diabetic rats by increasing DISC1 expression to promote mitophagy. This enhanced Aβ clearance, alleviating cytotoxicity in hippocampal neuronal cells.

## Background

The International Diabetes Federation (IDF) predicts that 783 million adults with diabetes will be diagnosed by 2045 [1]. Diabetes mellitus (DM) is often accompanied by complications, such as cognitive impairment. People with high blood sugar may have a higher risk of cognitive decline [2].

Synaptic plasticity regulates learning and memory in patients with type 2 diabetes [3, 4]. Amyloid-β (Aβ) peptide deposition and hyperphosphorylation of microtubule-associated protein tau have been reported in brain tissues of DM patients [5]. Moreover, Aβ deposition in the brain leads to neuronal toxicity, affecting microcirculation [6].

Mitophagy regulates mitochondria homeostasis and impairs mitophagy, contributing to neurodegenerative diseases. Moreover, hippocampal Aβ deposition impairs mitochondrial biogenesis. Therefore, autophagy and mitochondrial structure alterations are major causes of neuronal dysfunction [7]. In addition, disrupted mitochondrial homeostasis can cause cognitive dysfunction in diabetic patients. Thus, upregulated autophagy might protect against neurodegenerative diseases [8].

Disrupted-in-Schizophrenia1 (DISC1) is a novel mitophagy receptor that regulates neuronal function [9–11]. Downregulation of DISC1 results in mitochondrial dysfunction and impairs synaptic plasticity, causing cognitive decline [12]. Yang et al. [13] demonstrated that Disc1 knockdown in mice decreased recognition memory.

Currently, there are no effective drugs for diabetes-associated cognitive impairment. Acupuncture and moxibustion have shown good treatment effects against cognitive disorders [14, 15]. Electroacupuncture application (EA) at Zusanli (ST36) and Yishu (EX-B3) can decrease inflammatory cytokines levels, thereby improving learning and memory function in diabetic rats with cognitive disorders [16]. At the Baihui (GV20), EA contributes to the neuroprotection against CUMS by enhancing BDNF expression and improving hippocampal neurogenesis [17]. At both Baihui (DU20) and Dazhui (DU14), EA significantly reduces infarct volume and alleviates neuronal injury [18].

However, EA mechanisms have not been fully clarified. Herein, we explored the effects of electroacupuncture on learning and memory functions in STZ-treated rats fed on a high-fat diet. Electroacupuncture treatment promoted mitophagy by enhancing DISC1 expression to suppress Aβ toxicity.

## Methods

### Experimental animals and groups

Specific pathogen-free (SPF) male rats (*n* = 100; 3 months; 200 ± 20 g) were obtained from the Anhui Center of Laboratory Animals [certificate number: SCXK (Su) 2017-0003]. All rats were kept in a sterile room at 22 ± 3 °C and humidity of 55 ± 10%. They were provided with food and water ad libitum for 1 week. Subsequently, rats were randomized into two groups: control (*n* = 8) and model (*n* = 92). Model rats were fed on a high-fat, high-sugar diet for 30 days and intraperitoneally injected with STZ (25 mg/kg) [19]. After 72 h, tail vein blood was collected to determine random blood glucose levels (≥ 16.7 nmol/L represented diabetes in rats) [20]. Seventy-two rats were successfully modeled. Cognitive disorders were examined using the Morris water maze. The mean escape latency, and difference ratio between the model and control groups were calculated. A value >20% indicated successful modeling of cognitive impairment [21]. These rats were randomized into four groups: model (*n* = 8), Electroacupuncture application (EA) (*n* = 8), autophagy (rapamycin; *n* = 8), and EA+3-methyladenine (3-MA; *n* = 8). All experiments were carried out according to the “Guiding Opinions on Treating Laboratory animals” (Ministry of Science and Technology). All rats were anesthetized with intraperitoneal 0.3% sodium pentobarbital (30 mg/kg).

In the EA group, rats were first fixed and kept awake. The target sites were Yishu (EX-B3), Zusanli (ST36), BaiHui (GV 20), and Dazhui (DU14). Subcutaneous needles (size: 0.3525 mm; needle penetration depth: 4 mm) were used to probe Yishu and Zusanli. All puncture points were connected to Han's Electroacupuncture instrument. After reviewing the literature [22, 23], EA was performed using a stimulation current of 1 mA, frequency of 15 Hz, duration of 30 min, and once a day [14].

The rapamycin group received rapamycin (2 mg/kg) intraperitoneally once daily, and the rest of the treatment was identical. Rapamycin has been shown to induce autophagy [24].

The EA+3-MA group received 3-MA (1.5 mg/kg) intraperitoneally once daily (30 min after EA), and the rest of the treatment was identical. Different from rapamycin, 3-MA can inhibit autophagy [25].

Rats were treated for 4 weeks (1 day of rest after 6 days of intervention). The model and control groups were not treated.

### Experimental design

**Fig. 1:**
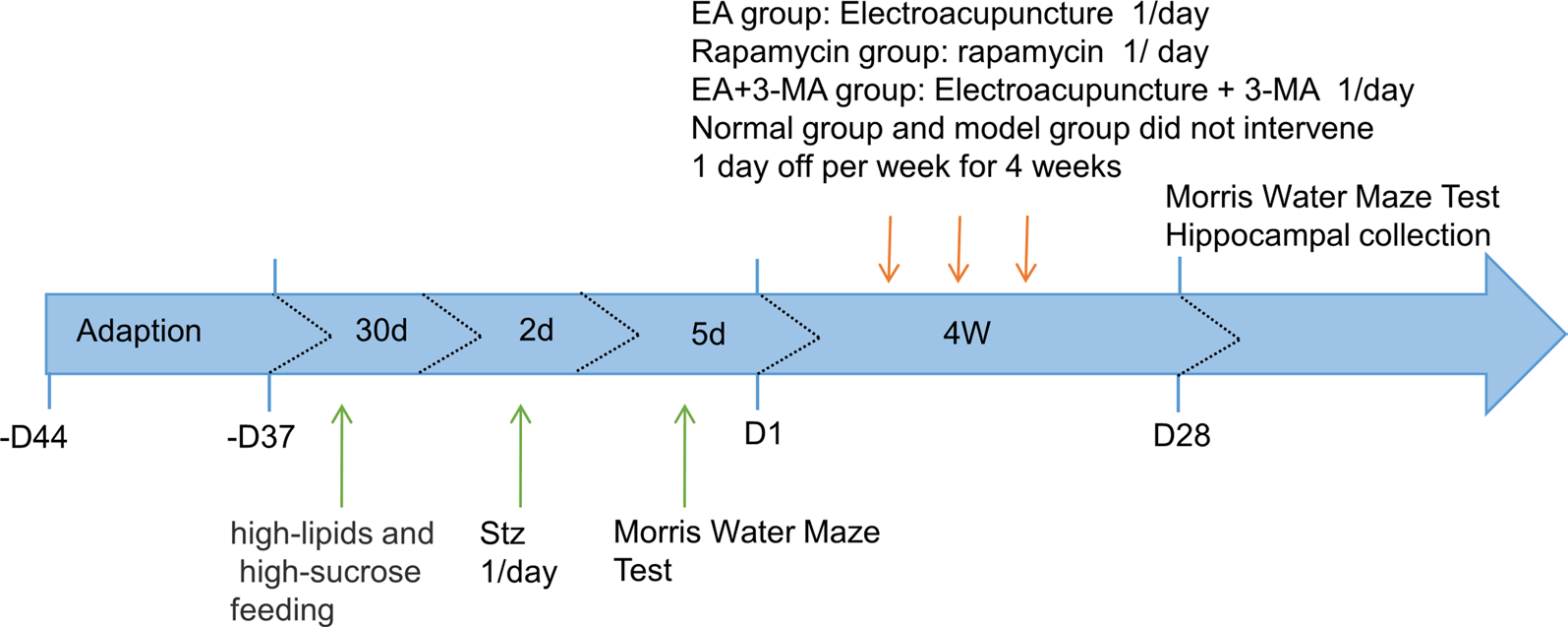
Experimental grouping roadmap

The experimental design is presented below (Fig. 1).

### Morris water maze

The Morris water maze test determined the learning and memory function after treatments. One day before the formal training, rats were placed in the pool and allowed to swim freely for 2 min to adapt to the new environment. The experiment included two learning tests: the place navigation test and the spatial probe test. (1) The navigation test was conducted to evaluate spatial learning. Rats were trained for two trials a day for four consecutive days. During the test, rats were positioned in the pool, facing the pool wall at the four starting locations. Each rat was allowed 2 min to find the hidden platform. Escape latency time was calculated as the time to find the hidden platform. If rats failed to reach the hidden platform within the 120 s, they were guided to the platform. After staying on the platform for 20 s, the escape latency time was recorded as 120 s. The crossing route to the platform was recorded with a video camera. (2) Memory was determined with a spatial test. Once the place navigation test was complete, the hidden platform was eliminated, and well-trained rats were permitted to swim for 2 min. The frequency of entering the hidden platform and percentage of distance traveled, defined as the distance traveled in the target quadrant divided by the distance traveled across all quadrants, were recorded within 120 s.

### Hematoxylin–eosin (H&E) staining

Paraformaldehyde (4%) was used to fix hippocampal tissues before paraffin-embedded. They were sliced into 5 µm sections and fixed on glass slides. Tissue morphology was examined after H&E staining. Images were captured with a microscope (Olympus BX53).

Rats were intraperitoneally anesthetized with 0.3% sodium pentobarbital (30 mg/kg), then fixed on the operating table (supine position). After disinfection, the thorax of the rats was cut open, and perfusion was performed from the heart. Once the rats became stiff, the heads were cut off and harvested on an ice plate. Rats' cerebral cortex and hippocampus were removed for alcohol dehydration and transparent with xylene. The paraffin wax (60 °C) was dipped three times and embedded into paraffin blocks. The wax blocks were sectioned with a microtome, the tissue pieces were placed in a water bath (40 °C) for layering, and slides were inserted at an angle to collect the sections. Sections were attached to slides in the oven (60 °C, 3 h). A series of xylene [xylene (i) 20 min, (ii) 20 min, and (iii) 20 min], ethanol [anhydrous ethanol (i) 5 min and (ii) 5 min], and alcohol (95% 5 min, 90% 5 min, 80% 5 min, and 70% 5 min) was applied. Next, they were stained with Mayer's hematoxylin solution and eosin. Slices were dehydrated with ethanol and sealed with neutral gum. Finally, the morphological changes of hippocampal cells were observed under a microscope.

### Enzyme-linked immunosorbent assay (ELISA)

First, rats were anesthetized with intraperitoneally-injected 0.3% sodium pentobarbital (30 mg/kg) and decapitated. The collection of hippocampal tissues was performed on ice, homogenized with a homogenizer, and centrifuged for 10 min at 10,000 rpm at 4 °C. Next, 1% of lysates were subjected to ELISA following the manufacturer’s instructions. The expression levels of Aβ_1-42_ were evaluated using a microplate reader at 450 nm.

### Sample preparation for electron microscopy

Briefly, 1 mm × 1 mm × 1 mm pieces of the hippocampal CA1 region were fixed in 2.5% glutaraldehyde phosphate buffer (4 °C, pH 7.4) for 4 h, then dehydrated with ethanol and acetone. They were embedded and cut into 60 nm sections for staining with lead citrate for 5–6 min and uranyl acetate for 20 min. Imaging was conducted by TEM (JEM1230).

### Immunofluorescence and immunohistochemistry staining

Rats from all groups were anesthetized by intraperitoneal injection of isoflurane. The chest cavity was quickly opened to expose the heart. Then, 0.9% NaCl solution was administered with a needle into the apex of the myocardium to flush blood from the vasculature. A small incision was cut on the right atrial appendage to observe the outcomes. After standard cardiac perfusion, the prefrontal hippocampus was fixed with 4% paraformaldehyde. Tissues were sliced into 3 µm sections, immersed thrice in xylene and a series of ethanol solutions, and rinsed with running water. Sections were incubated with goat serum for 0.5 h after antigen retrieval, then incubated for 1 h at 37 °C in the presence of primary antibodies (COXIV: 1:100; Lamp2: 1:100), and washed three times using 1× PBS buffer. Next, samples were incubated with fluorophore-conjugated secondary antibodies for 30 min at 37 °C in the dark. Slices were mounted using anti-fading reagents. Images were captured by fluorescence microscopy to determine the mean fluorescence of target proteins.

### Western blotting

Proteins were extracted from hippocampal tissues using RIPA lysis buffer. The concentration of extracted proteins was determined with a BCA protein assay kit. Equal protein amounts were separated on an SDS-PAGE gel, transferred onto polyvinylidene fluoride membranes for blocking for 2 h with 5% non-fat milk, and incubated with the following primary antibodies overnight at 4 °C: GAPDH: 1:1000 LC3: 1:1000; BCL2: 1:1000; P62: 1:1000; Beclin1: 1:2000; DISC1: 1:1000. After washing with 1× TBST buffer, membranes were incubated with HRP conjugated secondary antibodies (1:5000) for 120 min at room temperature (RT). The enhanced chemiluminescence reagent was used to develop protein blots, which were analyzed with Image J software.

### Reverse transcription-quantitative polymerase chain reaction (RT-qPCR)

Total RNAs from 100 mg hippocampus tissues were extracted using Trizol reagent and reverse-transcribed into cDNA with the HiScript III 1st Strand cDNA Synthesis Kit. The thermocycling conditions were: 25 °C/5 min, 50 °C/15 min, 85 °C/5 min, and 4 °C/10 min. DISC1 expression was examined using PowerUp™ SYBR^®^ Green. The thermocycling conditions were: 95 °C/10 min; 40 cycles of 95 °C/15 s and 60 °C/60 s; 95 °C/15 s, 60 °C/60 s, and 95 °C/15 s. The internal control in this assay was β-actin. Relative expressions of DISC1 mRNA were determined using the 2^−ΔΔCt^ method. The primers used in this assay are presented in Table 1.

**Table 1:** List of primers

Gene	Primer	Sequence (5′–3′)	PCR products (bp)
β-Actin	Forward	CACGATGGAGGGGCCGGACTCATC	240
	Reverse	TAAAGACCTCTATGCCAACACAGT	
Rat DISC1	Forward	CACTCGACCTGGCTGTTAGA	193
	Reverse	GATGACACGGCCCAAATCTC	

### Statistical analysis

Data are shown as means ± standard deviations (SDs) and were analyzed using SPSS 27.0. Multiple groups were compared by one-way analysis of variance (ANOVA). The mean values of two groups were compared with Fisher's least significant difference (LSD) method. A *p* ≤ 0.05 was considered statistically significant.

## Results

### Effects of electroacupuncture on body weight and blood glucose levels in diabetic rats

**Fig. 2:**
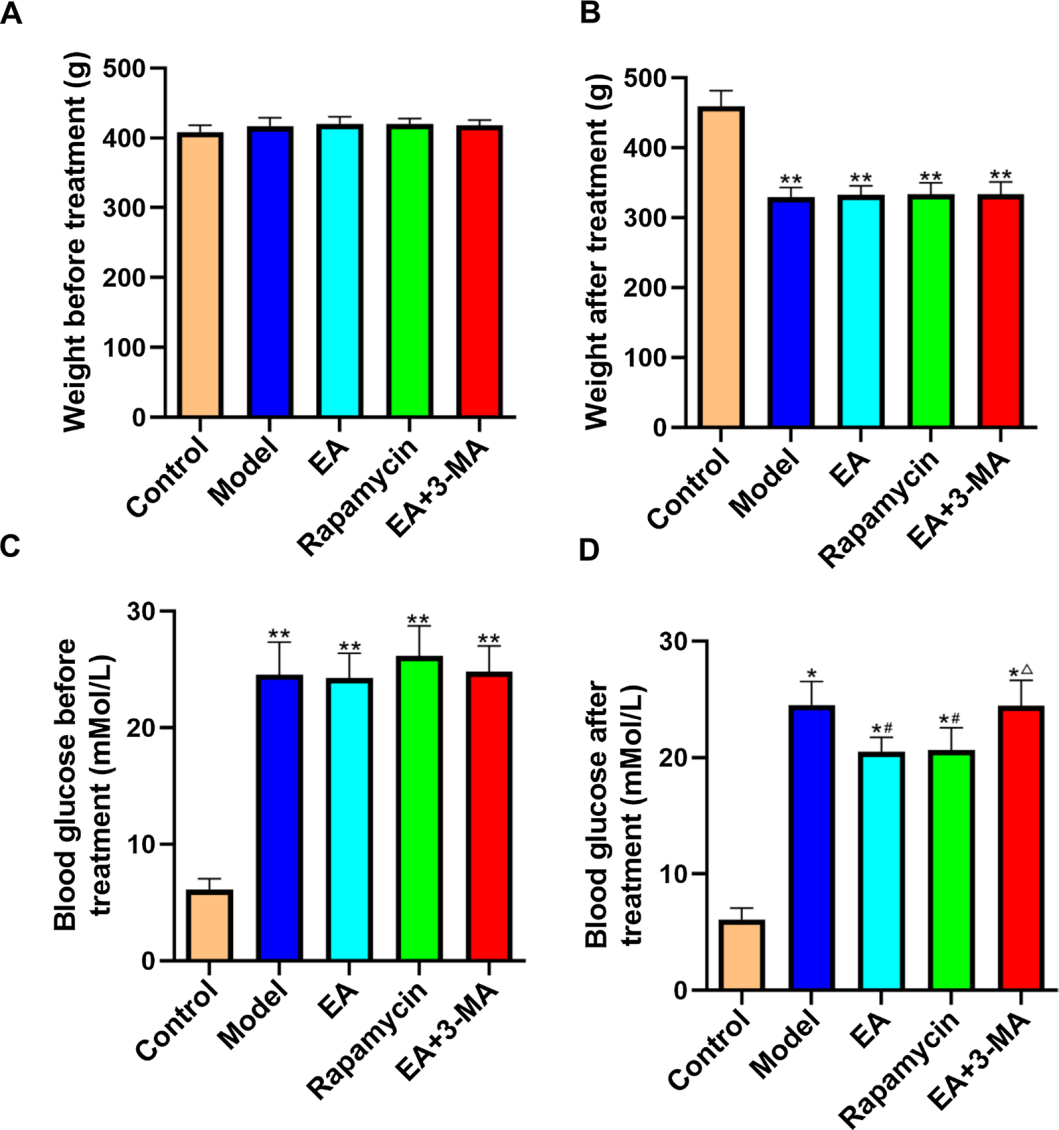
Effect of EA on body weight and blood sugar in diabetic rats. **a** Weight before treatment, **b** weight after treatment, **c** blood glucose before treatment, **d** blood glucose after treatment. Data are expressed as mean ± standard error of the mean (*n* = 8 in each group). *p < 0.05, **p < 0.01 versus control group; ^#^*p* < 0.05 versus model group; ^△^*p* < 0.05 versus EA group

Before treatment, the body weights did not differ between groups (Fig. 2a). The body weight was lower in the model, EA, Autophagy, and EA+3-MA groups compared to the control group (*p* < 0.01, Fig. 2b). Meanwhile, blood sugar was higher in the model, EA, Rapamycin, and EA+3-MA groups compared to the control group (*p* < 0.01, Fig. 2c). After EA treatment, blood sugar levels were lower in EA and Rapamycin groups compared to the model group (*p* < 0.01, Fig. 2d). Moreover, compared to the EA group, blood sugar was elevated in the EA+3-MA group (*p* < 0.05).

### Effects of EA on cognitive function in diabetic rats

**Fig. 3:**
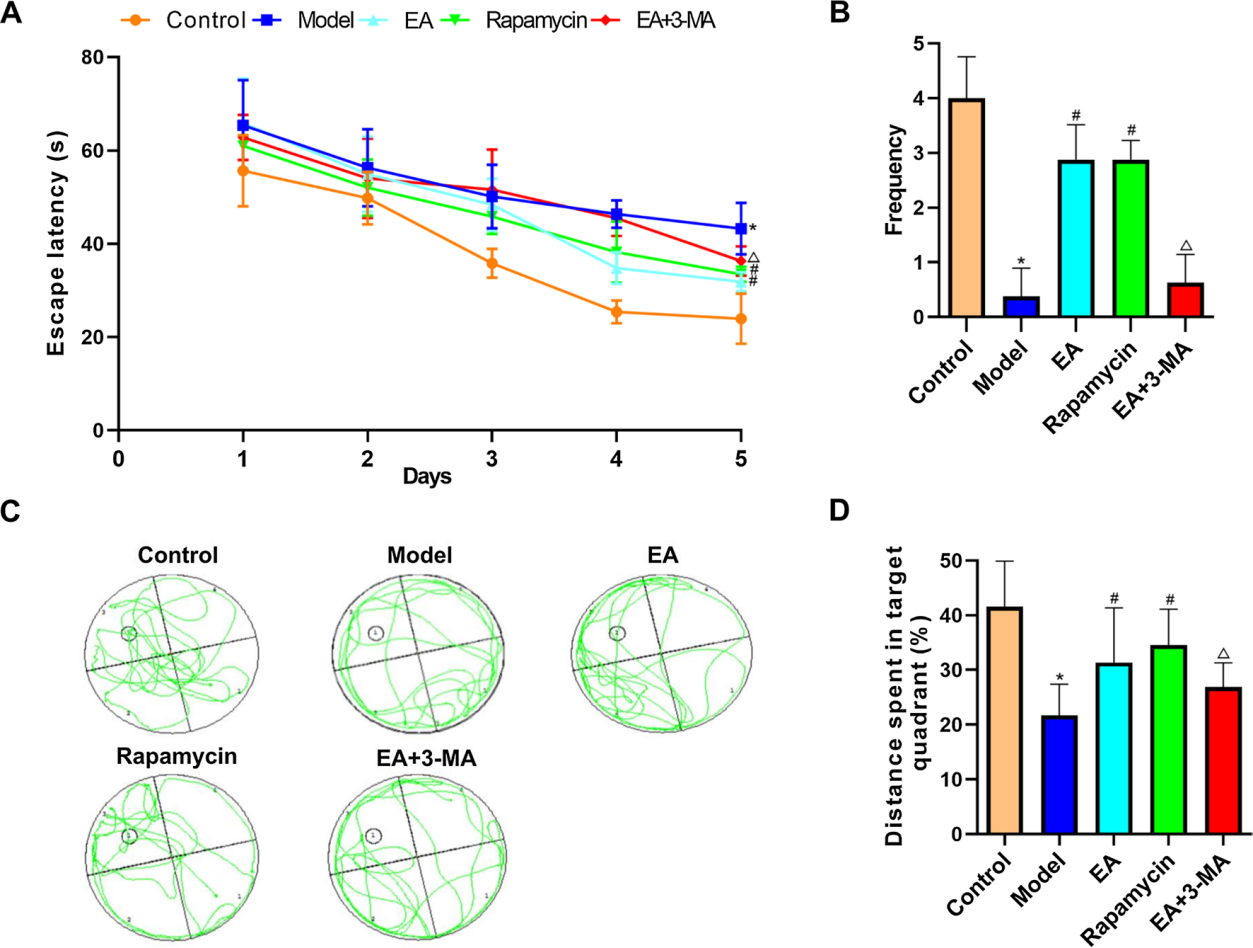
Effect of EA on learning and memory in diabetic rats with cognitive impairment by the Morris water maze test. **a** Escape latency, and **b** swimming trajectories were recorded to assess the learning ability. **c **Number of platform crossings and **d** percentage of distance that the rats in the target quadrant. All data are expressed as means ± standard error. *n* = 8 rats per group. *p < 0.05 versus control group; #*p* < 0.05 versus model group; ^△^*p* < 0.05 versus EA group

During the five training days, all groups presented significantly reduced escape latency time in the Morris water maze experiment (Fig. 3a). Model group rats spent more time finding the hidden platform compared to the control group rats (*p* < 0.05). On day 3, rats in EA, Rapamycin, and EA+3-MA groups took less time to find the target than control rats (*p* < 0.05). Rats in EA and rapamycin groups found the hidden platform faster than those in the model group on days 4 and 5 (*p* < 0.05). Moreover, compared to the EA group, rats in the EA+3-MA group spent longer to find the target (Fig. 3b). Furthermore, the spatial probe test w showed that the frequency of entering the hidden platform was markedly lower in the model group compared to the control group. Similarly, the counts of platform crossings in EA and rapamycin groups were higher compared to the model group (*p* < 0.05). Rats in the EA+3-MA group showed a reduced number of platform crossings compared to the EA group (*p* < 0.05; Fig. 3c). Compared to control rats, the percentage of distance traveled was lower for the model group (*p* < 0.05). Consistently, rats in the EA and rapamycin groups had a higher percentage of distance traveled than model group rats (*p* < 0.05). The 3-MA treatment decreased the percentage of distance traveled compared to the EA group (*p* < 0.05; Fig. 3d).

### Effects of electroacupuncture on the hippocampal CA1 area morphology

**Fig. 4:**
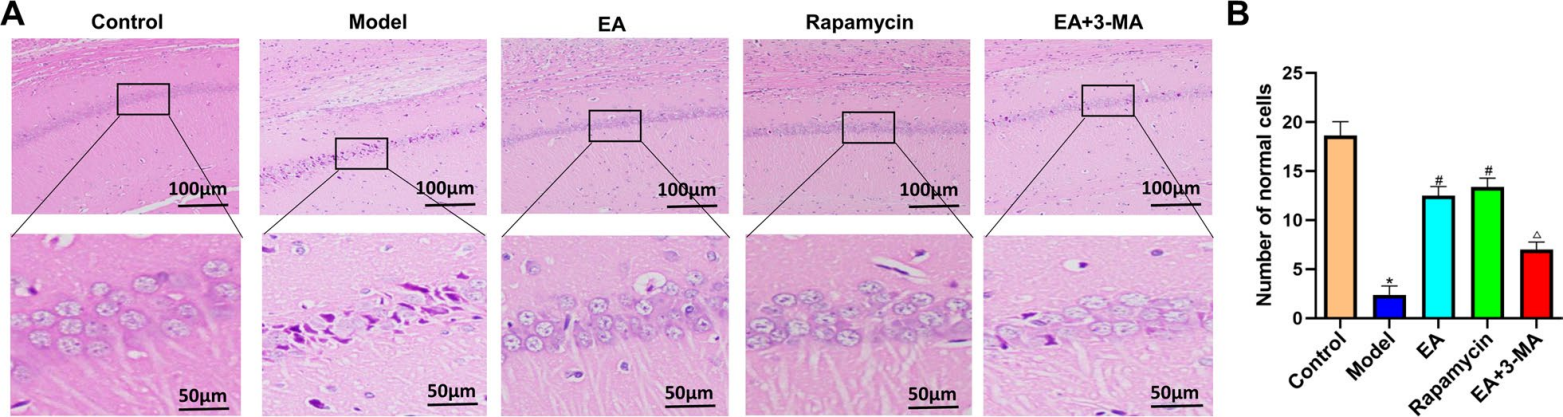
Effects of EA on morphological changes in the hippocampal CA1 area. **a** HE staining of hippocampal CA1 area. Upper lane, Scale bar = 100 μm, magnification ×200. (the inside area of the red black rectangle. Scale bar = 50 μm, magnification ×400). The pyramidal neurons in the CA1 area of the hippocampus controls had spherical and plump nuclei, clear nucleoli, and cytoplasm. The model group hippocampus neurons were loosely packed, fusiform or polygonal in shape, with irregular nuclei. Some of the neurons displayed karyopyknosis, hyperchromatic cytoplasm, and fuzzy or missing nucleoli. These morphological alterations were greatly improved in the EA, rapamycin, and EA+3-MA groups, with the EA group showing the greatest improvement. **b** Comparison of the number of normal cells in all groups. Data are expressed as mean ± standard error of the mean (*n* = 8 in each group). *p < 0.05 versus control group; ^#^*p* < 0.05 versus model group; ^△^*p* < 0.05 versus EA group

Furthermore, H&E staining was used to identify the impacts of EA on hippocampus neuron morphology. The CA1 hippocampal neurons of controls were morphologically normal (Fig. 4a), with round and plump nuclei. The hippocampal CA1 region neurons were disorganized and morphologically aberrant after modeling, while the EA, rapamycin, and EA+3-MA groups had similar degrees of disorder in cell organization and better morphology. Moreover, modeling decreased the number of normal cells compared to the controls (*p* < 0.05). The EA and rapamycin group had more normal cells than the model group (*p* < 0.05). The EA+3-MA group had fewer normal hippocampal cells than the EA group (*p* < 0.05) (Fig. 4b).

### Effects of electroacupuncture on Aβ1-42 expression of the hippocampus in diabetic rats

**Fig. 5:**
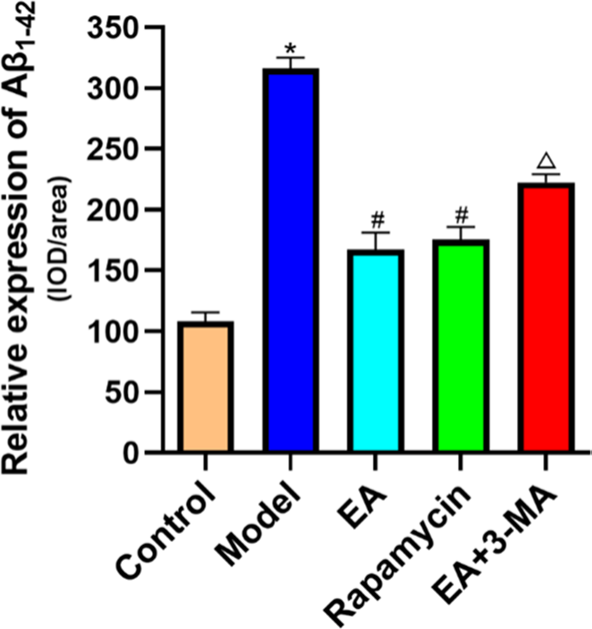
Effect of EA on Aβ1-42 expression. Data are expressed as mean ± standard error of the mean (*n* = 8 in each group). *p < 0.05 versus control group; ^#^*p* < 0.05 versus model group; ^△^*p* < 0.05 versus EA group

The model group had higher Aβ1-42 levels in the hippocampus than controls (*p* < 0.05). The EA and rapamycin group also had lower levels of Aβ1-42 in the hippocampus CA1 region than the model group (*p* < 0.05). Compared to the EA group, 3-MA therapy increased V levels in the hippocampus (*p* < 0.05; Fig. 5).

### Effects of electroacupuncture on the ultrastructure of hippocampal neurons in diabetic rats

**Fig.6:**
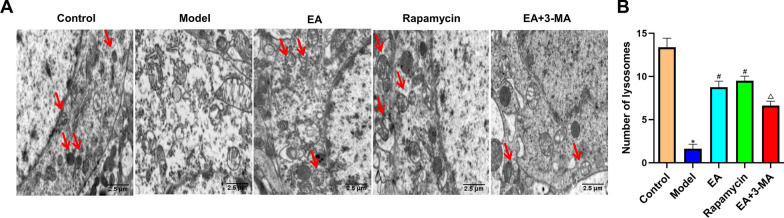
Effect of EA on ultrastructure of hippocampal neurons in diabetic rats. **A** Electron microscopic pictures of hippocampal neurons in diabetic rats. Scale bar = 1 μm, magnification ×6000. The protective effects of EA on ultrastructural damage in the hippocampus CA1 area of diabetic rats were identified by electron microscopy. **B** Comparison of the number of autophagic vacuoles in diabetic rats. Data are expressed as mean ± standard error of the mean (*n* = 8 in each group). *p < 0.05 versus control group; ^#^*p* < 0.05 versus model group; ^△^*p* < 0.05 versus EA group

Diabetic rats presented fewer autophagosomes and lysosomes than controls, as well as a broken nuclear envelope, an uncertain structure of the double membrane nuclear envelope, uneven chromatin distribution, and damaged mitochondrion. The EA and rapamycin group had less damage than the model group, with more organelles and autophagic vacuoles, less irregular and atrophied organelles, and a smoother nuclear envelope. The 3-MA therapy altered the ultrastructure of hippocampus neurons more than in the EA group. The TEM showed a muddled nuclear membrane, plenty of irregular organelles, and few autophagic vacuoles (Fig. 6a). Moreover, compared to controls, modeling reduced autophagic vacuoles (*p* < 0.05). The EA and rapamycin group had more autophagic vacuoles than the model group (*p* < 0.05). The EA+3-MA group had fewer autophagic vacuoles than the EA group (*p* < 0.05; Fig. 6b).

### Effects of electroacupuncture on hippocampal mitochondrial rapamycin in diabetic rats

**Fig. 7:**
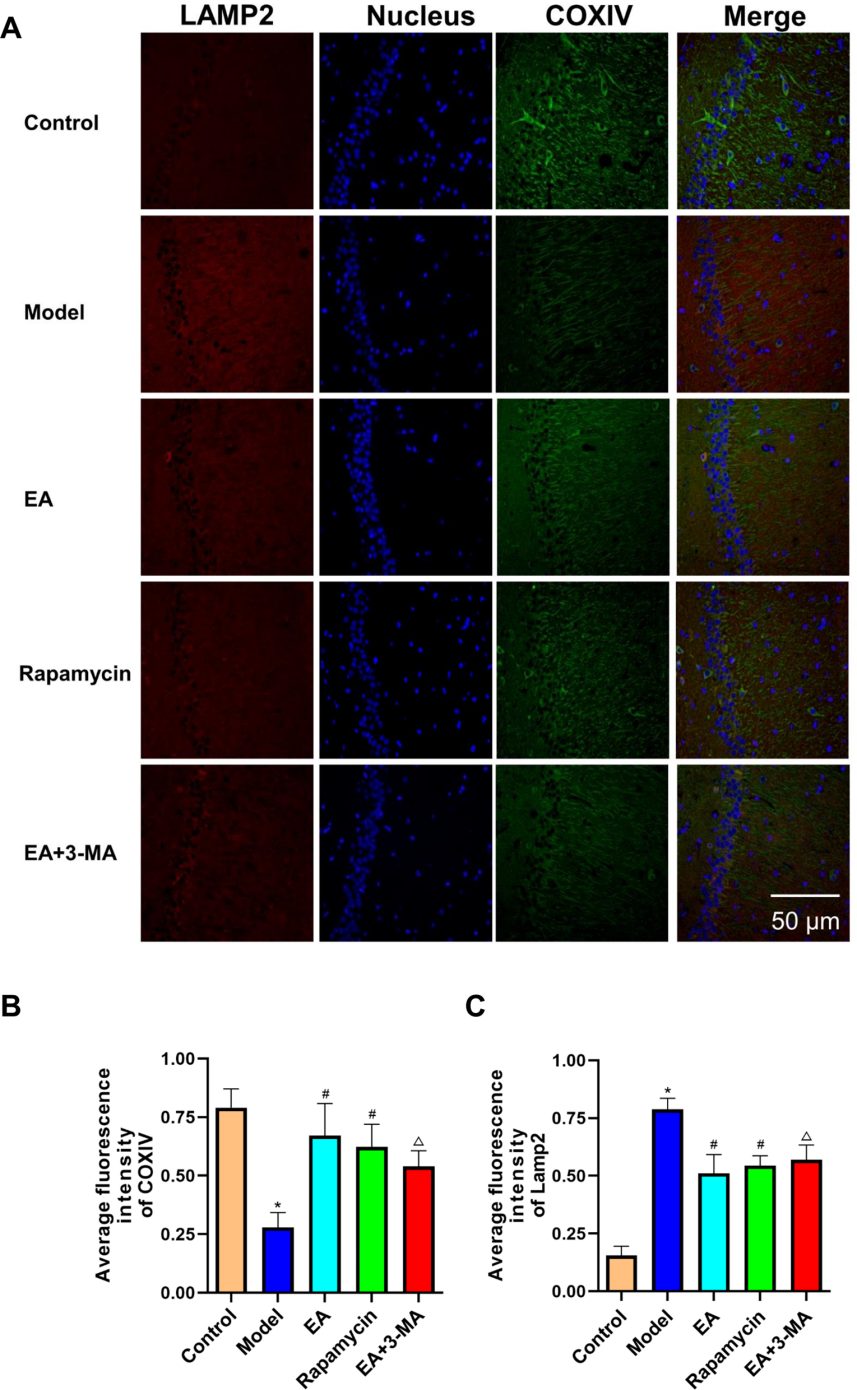
Comparison of immunofluorescence staining pictures of hippocampus in each group. Red shows Lamp2 protein expression, green shows COXIV expression, blue is the labeled nucleus. **a** Immunofluorescence pictures of Lamp2 and COXIV of rats in each group magnification ×400. **b** Average fluorescence intensity of Lamp2 of rats in each group. **c** Average fluorescence intensity of COXIV of rats in each group. *p < 0.05 versus control group; ^#^*p* < 0.05 versus model group; ^△^*p* < 0.05 versus EA group

The LAMP2 signal density and COXIV fluorescence intensity were markedly higher in the model group than in controls (*p* < 0.05; Fig. 7). Less disordered nuclei were observed in the EA and rapamycin groups than in the model group. The LAMP2 fluorescence intensity was lower, and COXIV fluorescence intensity was higher in the EA and rapamycin groups than in the model group (all *p* < 0.05). Moreover, EA-treated rats had more disordered nuclei after 3-MA therapy. The 3-MA administration increased LAMP2 and decreased COXIV fluorescence compared to the EA group (*p* < 0.05).

### Effects of electroacupuncture on the expression of autophagy-related proteins in hippocampus tissues of diabetic rats

DISC1, Beclin1, LC3, P62, and BCL2 proteins are essential molecules that regulate autophagy development and progression. They jointly regulate the generation of autophagosomes, and P62 is involved in autophagy degradation. The LC3-II to LC3-I ratio is currently recognized as an important marker of autophagy activation [26, 27]. Because these factors are closely related to the activation and activity of autophagy, their expression levels in DCI patients are often used to infer autophagy activity.

**Fig. 8:**
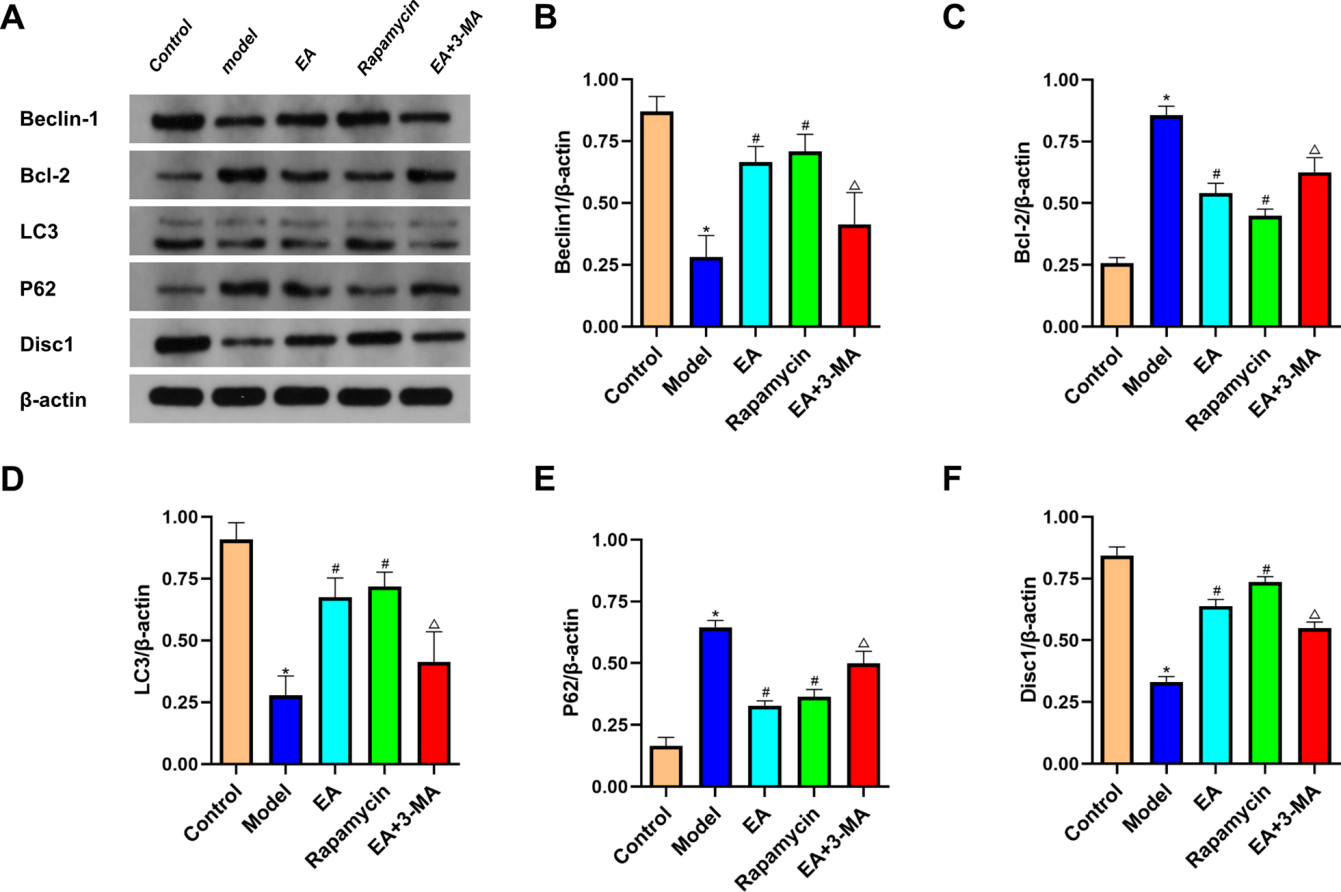
Effect of EA on autophagy related proteins in diabetic rats. **a** Protein bands of Beclin1, Bcl-2, LC3-II/I, P62, Disc1 and β-actin. **b** Quantitative analysis of Beclin1 relative expression. **c** Quantitative analysis of Bcl-2 relative expression. **d** Quantitative analysis of LC3-II/I relative expression. **e** Quantitative analysis of P62 relative expression. **f** Quantitative analysis of Disc1 relative expression. Data are expressed as mean ± standard error of the mean (*n* = 8 in each group). *p < 0.05 versus control group; ^#^*p* < 0.05 versus model group; ^△^*p* < 0.05 versus EA group

The modeling reduced the expression of DISC1, Beclin1, and LC3-II/I, while P62 and BCL2 increased (both *p* < 0.05; Fig. 8a). The EA and rapamycin group also had higher levels of DISC1, Beclin1, and LC3-II/I and lower levels of P62 and BCL2 than the model group (both *p* < 0.05). Compared to the model group, 3-MA therapy lowered DISC1, Beclin1, and LC3-II/I, while P62 and BCL2 increased (Fig. 8).

### Effect of electroacupuncture on Disc1 gene expression in diabetic rats

**Fig. 9:**
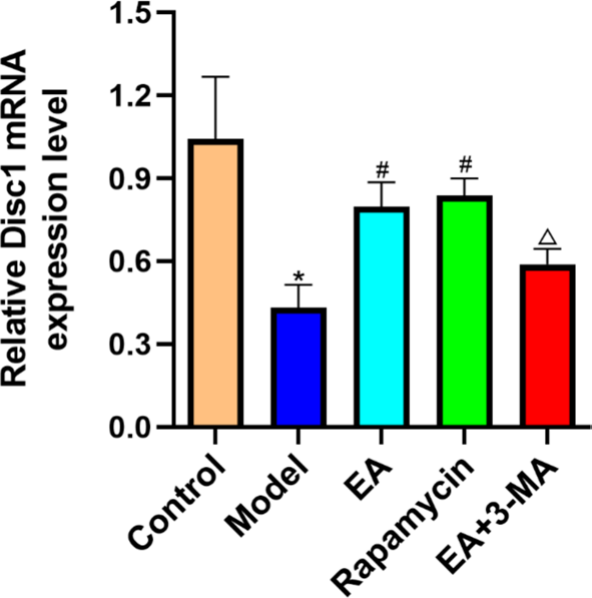
Effect of EA on the mRNA expression of Disc1 in diabetic rats determined by fluorescence quantitative PCR. Data are expressed as mean ± standard error of the mean (*n* = 8 in each group). *p < 0.05 versus control group; ^#^*p* < 0.05 versus model group; ^△^*p* < 0.05 versus EA group

The modeling reduced DISC1 expression compared to the controls (*p* < 0.05). The EA and rapamycin group had greater levels of DISC1 than the model group (*p* < 0.05). The 3-MA treatment lowered DISC1 levels more than the model group (*p* < 0.05; Fig. 9).

## Discussion

Herein, we showed that EA improved learning and function in diabetic model rats by promoting mitophagy and increasing DISC1 expression and Aβ clearance. The mechanisms by which EA improves cognitive function remain elusive [28]. Here, model group rats spent more time finding the hidden platform than control group rats. The EA treatment group decreased the time spent to find the target and increased the frequency of entering the hidden platform, indicating that EA improved the memory and learning function of diabetic rats.

Previous studies have suggested that electroacupuncture stimulation at “Baihui” (DU20) can improve learning and memory function in stroke patients [29]. In addition, electroacupuncture stimulation at “Baihui” and “Dazhui” showed good effects against cerebral ischemia [30, 31]. Moreover, EA at “Zusanli” (ST36) can suppress the expression of hyperphosphorylated tau protein in the hippocampus of diabetic rats to confer neuroprotection [32]. In addition, electroacupuncture at “Zusanli” (ST36), “Neiting” (ST44), and “Yishu” (EX-B3) can improve learning and memory function in diabetic rats [33] by suppressing the expression of p38 MAPK, p-p38 MAPK, STAT3, p-STAT3, IL-6, IL-1β, and TNF-α in the hippocampus [16]. Therefore, “Zusanli” (ST36), “Daizhui” (DU14), “Yishu” (EX-B3), and BaiHui (GV 20) are used to treat diabetes-associated cognitive decline. Hence, investigating the therapeutic efficacy of different acupuncture points on cognitive function is crucial.

The Aβ protein participates in cognitive impairment associated with type 2 diabetes (T2DM). Studies have shown that Aβ is a marker of cognitive impairment in T2DM [34]. Previous studies have shown that hippocampal deposition of Aβ resulted in abnormal mitochondrial dynamics leading to neuronal dysfunction [7]. However, whether mitophagy mediates the EA effects on learning and memory function is unclear. In the present study, the EA and rapamycin groups had lower Aβ levels, whereas the EA+3MA group had slightly lower Aβ expression than the model group, indicating that EA and rapamycin treatment attenuated Aβ-induced neuronal impairment. Since the Aβ expression level in the model and EA+3MA groups was similar, we speculated that EA conferred protection by promoting mitophagy.

Furthermore, the H&E staining revealed that neurons within the hippocampal CA1 region in the control group exhibited normal morphology, structural arrangement, and chromatin structures. In the model group, the hippocampal neurons were loosely arranged and disordered and exhibited irregular nuclei based on karyopyknosis, hyperchromatic chromatin, and karyolysis results. The cytoplasmic organelles were disorganized, and their number was reduced. Altogether, these results indicated that Aβ induced neuronal impairment. Compared to the model group, Aβ expression decreased in EA, rapamycin, and EA+3-MA groups, indicating that EA intervention reduced neuronal apoptosis to ameliorate neuronal damage in diabetic rats.

The ultrastructure of neurons in the hippocampus of controls was morphologically normal, with numerous mitochondria, autophagosomes, and lysosomes, even distribution of chromatin, evident nuclear double membrane structure, and intact nuclear envelope. The EA group also had more organelles and autophagic vacuoles, less atrophied organelles, and a smoother nuclear membrane. This set of neurons had more autophagic vacuoles, damaged mitochondria and endoplasmic reticulum (ER), and partly uniform chromatin distribution. After 3-MA therapy, we detected atrophied and damaged organelles, fewer autophagic vacuoles, nuclear envelope collapse, and unclear nuclear envelope.

The DISC1 receptor plays an important role in neurotherapy [35–38]. Besides Beclin1, LC3-II is an autophagy substrate p62. The EA group had greater levels of DISC1, Beclin1, and LC3 than the model group, suggesting that the protective impact of EA intervention was mediated by mitophagy activation in the hippocampus. The expression of DISC1 was also elevated in the rapamycin and EA+3-MA groups compared to the model group. The EA and rapamycin groups had higher levels of DISC1. A reduction in DISC1 expression might affect mitochondrial function, inhibit synaptic plasticity, and ultimately impair learning and memory.

The real-time PCR results indicated a higher DISC1 expression in EA and rapamycin groups. Moreover, the 3-MA-treated group had lower DISC1 expression than the EA group, indicating that DISC1 downregulation impaired mitochondrial function, finally leading to impaired learning and memory function.

Rapamycin has been used to induce autophagy. We found that EA intervention had a similar effect to rapamycin in diabetes-associated cognitive disorders. The expression patterns of mitophagy proteins DISC1, Beclin1, and LC3 were consistent. Meanwhile, EA treatment decreased the expression of these proteins in 3-MA-treated rats. Hence, EA is a potent inducer of rapamycin.

In a previous study, male mice bilaterally injected into the hippocampus before water maze training showed no alteration in learning ability. However, significant damage was observed in the hippocampus 24 h after injection [39]. Studies have shown that 3-MA injection can inhibit hippocampal autophagy and alleviate cognitive impairment [40]. Additional administration of 3-MA inhibited cognitive dysfunction in mice [41]. However, the effects of 3-MA alone on learning and memory in rats were not explored here and should be in future studies.

The therapeutic benefit of EA against diabetes-associated cognitive impairment has not been clarified. The pathological mechanisms of diabetes-associated cognitive impairment are extremely complex. Hyperphosphorylation of tau protein has been linked to the development of diabetes-associated cognitive disorders. Given that several ACU points might exert similar therapeutic effects, it is unclear which ones are responsible for the therapeutic benefits of acupuncture. Therefore, future studies should investigate the molecular mechanisms underlying diabetes-induced cognitive impairment in diabetic rats.

## Conclusions

In summary, EA treatment improved the learning and memory function of diabetic model rats by promoting mitophagy, inducing DISC1 expression, and Aβ clearance from hippocampal neuronal cells.

## Data Availability

The data sets generated and/or analysed during the current study are not publicly available due [REASON WHY DATA ARE NOT PUBLIC] but are available from the corresponding author on reasonable request.

## Abbreviations

DM: Diabetes mellitus
STZ: Streptozotocin
Aβ: Amyloid-β
DISC1: Disrupted-in-Schizophrenia 1
EA: Electroacupuncture
